# Volatile Anesthesia in Contemporary Cardiac Surgery: Clinical Implications, Organ Protection and Perspectives for Personalized Perioperative Care

**DOI:** 10.3390/jpm16030138

**Published:** 2026-03-01

**Authors:** Debora Emanuela Torre, Carmelo Pirri

**Affiliations:** 1 Department of Cardiac Anesthesia and Intensive Care Unit, Cardiac Surgery, Ospedale dell’Angelo, Mestre, 30174 Venice, Italy; 2 Department of Neurosciences, Institute of Human Anatomy, University of Padova, 35122 Padua, Italy; carmelo.pirri@unipd.it

**Keywords:** inhalational anesthesia, volatile anesthesia, sevoflurane, desflurane, cardiac surgery, cardiopulmonary bypass, ischemia-reperfusion injury, ischemic preconditioning

## Abstract

**Background**: Interest in inhalational anesthesia in cardiac surgery has resurged as volatile anesthetics exert biological effects extending beyond hypnosis. Sevoflurane and desflurane activate mitochondrial cytoprotective signaling pathways, modulate inflammatory and endothelial responses and may attenuate ischemia–reperfusion injury during cardiopulmonary bypass, potentially influencing postoperative organ function and recovery. **Methods**: This narrative review critically examines experimental and clinical evidence on the use of volatile anesthetics in cardiac anesthesia. The current literature was analyzed to elucidate mechanistic foundations of myocardial and extracardiac organ protection, hemodynamic and metabolic effects, and the influence of patient-specific vulnerability profiles on perioperative outcomes. **Results**: Preclinical studies consistently demonstrate that volatile anesthetics trigger mitochondrial protective pathways, including K-ATP channel activation, controlled reactive oxygen species signaling and inhibition of the mitochondrial permeability transition pore. Clinical studies suggest potential benefits in myocardial protection and modulation of systemic inflammatory and microcirculatory responses. However, translation into consistent clinical outcome improvement remains heterogeneous, influenced by variability in surgical procedures, anesthetic protocols and patient risk stratification. **Conclusions**: Volatile anesthetics exhibit mechanistic properties supporting a potential role in organ protection during cardiac surgery. Nevertheless, clinical evidence remains inconclusive, underscoring the need for refined patient stratification and precision-based perioperative strategies. Identifying knowledge gaps and research priorities may facilitate rational, individualized integration of inhalational anesthesia into contemporary cardiac surgical practice.

## 1. Introduction

Cardiac surgery represents one of the most physiologically challenging settings in contemporary perioperative medicine. Despite substantial advances in surgical techniques, cardiopulmonary bypass technology and postoperative critical care, ischemia–reperfusion injury and systemic inflammatory activation remain central determinants of myocardial dysfunction and multiorgan impairment after cardiac procedures [1,2]. Within this complex pathophysiological landscape, anesthetic management has progressively evolved from a purely supportive role toward a potential modulator of perioperative cellular stress responses. Volatile anesthetics have long been integral to cardiac anesthesia because of their titratability and favorable hemodynamic profile. However, experimental observations over the past two decades have suggested that these agents exert biological effects extending beyond hypnosis and cardiovascular control [3,4]. Sevoflurane, desflurane and isoflurane have been shown to engage intracellular signaling networks implicated in endogenous cytoprotection, raising the possibility that anesthetic choice may influence susceptibility to ischemia–reperfusion injury [5]. This concept, often referred to as anesthetic-induced preconditioning, has prompted renewed scientific and clinical attention to inhalational anesthesia in cardiac surgery [6,7,8,9]. Initial clinical studies reported reductions in biochemical markers of myocardial injury and suggested potential improvements in early postoperative outcomes [4,10,11,12,13,14,15,16,17,18,19,20,21,22]. Subsequent investigations, however, have yielded heterogeneous results, reflecting variability in surgical complexity, anesthetic protocols, cardioprotective adjuncts and baseline patient risk [23,24,25,26,27,28,29,30,31]. Consequently, the true magnitude and clinical relevance of volatile anesthetic-mediated organ protection remain incompletely defined. Concurrently, perioperative medicine is undergoing a paradigm shift toward precision-based strategies that account for interindividual variability in physiological reserve, comorbid burden and molecular response to injury. Within this emerging framework, the possibility that specific patients subgroup may preferentially benefit from volatile anesthetic-mediated cytoprotection has regained relevance. This narrative review examines the mechanistic foundations and clinical evidence supporting the use of volatile anesthetics in contemporary cardiac surgery. Particular attention is given to myocardial and extracardiac organ protection, interactions with cardiopulmonary bypass-induced systemic responses and the potential integration of inhalational anesthesia into personalized perioperative management strategies. This review integrates mechanistic cardioprotection, extracardiac organ effects, cardiopulmonary bypass interactions and sustainability considerations within a precision perioperative medicine framework, providing a contemporary perspective to inform anesthetic decision-making in cardiac surgical practice.

## 2. Materials and Methods

This narrative review was developed through a targeted and critical appraisal of experimental and clinical literature addressing the role of volatile anesthetics in cardiac surgery. A structured search of PubMed, Web of Science and Scopus databases was conducted to identify relevant articles published in English up to January 2026. Search terms included combinations of “volatile anesthetics”, “sevoflurane”, “desflurane”, “cardiac surgery”, “ischemia-reperfusion injury”, “anesthetic preconditioning”. Original experimental studies, randomized clinical trials, observational studies, meta-analyses and authoritative reviews were considered eligible if they addressed mechanistic aspects of volatile anesthetic-mediated cytoprotection, hemodynamic metabolic effects in cardiac anesthesia or clinical outcomes in adult cardiac surgical populations. Studies focusing exclusively on non-cardiac surgery or non-volatile anesthetic techniques were excluded unless providing essential mechanistic or translational insights. Article selection was guided by relevance to contemporary cardiac surgical practice, methodological quality and contribution to understanding translational and clinical implications. Reference lists of key publications were manually screened to identify additional pertinent studies. Given the narrative and hypothesis-generating scope of this review, characterized by substantial heterogeneity in study design, patient populations, anesthetic protocols, cardioplegia strategies and outcome definitions, no formal meta-analytic synthesis was performed. Instead, evidence was qualitatively integrated to contextualize mechanistic plausibility, identify areas of clinical convergence and highlight persisting uncertainties relevant to precision perioperative medicine.

## 3. Results

### 3.1. Pathophysiological Basis of Ischemia–Reperfusion Injury in Cardiac Surgery

Myocardial ischemia and subsequent reperfusion remain inherent to most cardiac surgical procedures requiring cardiopulmonary bypass and aortic cross-clamping. Although contemporary myocardial protection strategies have substantially improved operative safety, reperfusion itself triggers a complex cascade of cellular events that contribute to contractile dysfunction and systemic organ injury [1,2,32,33]. At the cardiomyocyte level, abrupt restoration of oxygen delivery promotes mitochondrial calcium overload, excessive generation of reactive oxygen species and opening of the mitochondrial permeability transition pore, leading to loss of membrane potential, ATP depletion and initiation of apoptotic and necrotic pathways. These intracellular processes translate into myocardial stunning, impaired diastolic relaxation and vulnerability to arrhythmogenesis in the early postoperative period [34,35,36,37]. Beyond the heart, cardiopulmonary bypass induces a systemic inflammatory response characterized by complement activation, cytokine release, endothelial dysfunction and microcirculatory impairment. Disruption of endothelial glycocalyx integrity and leukocyte–endothelial interactions further amplify capillary leak and tissue edema, thereby contributing to pulmonary dysfunction, acute kidney injury and neurological complications [38,39,40]. The magnitude of ischemia–reperfusion and inflammatory injury is modulated by procedural factors, cardioplegia strategies, temperature management and individual patient susceptibility, underscoring the multifactorial nature of postoperative organ dysfunction [2,41,42,43,44]. Within this pathophysiological framework, interventions capable of modulating mitochondrial stress responses and systemic inflammatory activation have emerged as attractive targets for perioperative organ protection. Volatile anesthetics have been consistently shown to interact with several of these key pathways, providing a mechanistic rationale for their proposed protective role in cardiac surgical patients [45,46,47].

### 3.2. Mechanism of Volatile Anesthetic-Induced Cytoprotection

Experimental research over the past two decades has established that volatile anesthetics activate endogenous cellular defense programs that mirror classical ischemic preconditioning. Sevoflurane, isoflurane and desflurane interact directly with mitochondrial and sarcolemmal ion channels, initiating signaling cascades that enhance cellular tolerance to subsequent ischemic stress (Table 1). Among these mechanisms, the activation of ATP-sensitive potassium channels (K-ATP) plays a central role in stabilizing mitochondrial membrane potential, limiting calcium influx and reducing energy expenditure during hypoxic conditions [48,49,50,51]. Concurrently, controlled generation of reactive oxygen species functions as a second messenger, triggering downstream protein kinase pathways that reinforce cytoprotective gene expression and inhibit apoptotic signaling [52,53]. A pivotal target of volatile anesthetics-induced protection is the mitochondrial permeability transition pore, whose pathological opening at reperfusion represents a final common pathway of cellular death [34,54]. Experimental models consistently demonstrate that exposure to volatile anesthetics before or during ischemia delays pore opening, preserves mitochondrial integrity and sustains adenosine triphosphate synthesis. These effects translate into reduced infarct size, improved post-ischemic contractile recovery and attenuation of myocardial stunning [51,54,55]. Beyond cardiomyocyte-specific pathways, volatile anesthetics exert relevant systemic actions. They modulate endothelial nitric oxide signaling, attenuate leukocyte adhesion and dampen pro-inflammatory cytokine release, collectively preserving microcirculatory flow and limiting capillary leakage [56,57,58]. Emerging evidence also suggests interactions with glycocalyx stability and platelet function, potentially influencing coagulation and inflammatory cross-talk during cardiopulmonary bypass [59,60,61,62]. The timing of volatile anesthetics administrations appears critical. Protective effects have been described both when exposure precedes ischemia, consistent with anesthetic-induced preconditioning and, when delivered during early reperfusion, consistent with postconditioning phenomena [51,63,64]. However, the relative contribution of these temporal windows and their optimal clinical translation remain incompletely defined. Together, these mechanistic insights provide a coherent biological rationale for the proposed organ protective properties of volatile anesthetics in cardiac surgery and set the foundation for evaluating their clinical relevance.

### 3.3. Hemodynamic and Metabolic Effects in Cardiac Anesthesia

In addition to their cytoprotective properties, volatile anesthetics exert distinctive cardiovascular and metabolic effects that influence intraoperative management in cardiac surgery. Sevoflurane, desflurane and isoflurane produce dose-dependent reductions in systemic vascular resistance through smooth muscle relaxation and modulation of autonomic tone, resulting in afterload reduction that may facilitate ventricular ejection in patients with preserved contractile reserve. Simultaneously, direct myocardial depressant effects, mediated by altered calcium handling and beta-adrenergic responsiveness, necessitate careful titration in patients with impaired ventricular function or limited hemodynamic reserve [65,66,67]. Coronary vasodilation induced by volatile anesthetics enhances myocardial perfusion under normal conditions; however, in the setting of coronary artery disease, regional flow redistribution and potential steal phenomena remain theoretical concerns, although clinically relevant ischemia attributable to this mechanism appears uncommon in contemporary practice [68,69]. Heart rate modulation is generally modest, but may influence diastolic filling and myocardial oxygen balance in susceptible patients [65]. Metabolically, volatile anesthetics affect substrate utilization and mitochondrial efficiency [51]. Experimental data indicate that volatile anesthetics modulate mitochondrial energetics during ischemic stress, with preservation of mitochondrial function and high-energy phosphate availability [70,71]. Volatile agents may also impair perioperative glucose regulation through reduced insulin secretion and insulin resistance, a clinically relevant issue in cardiac surgery where insulin sensitivity changes dynamically [72]. By contrast, evidence that volatile anesthetic exposure, per se, meaningfully modulates lactate kinetics during cardiopulmonary bypass is limited and inconsistent [73,74]. Importantly, the hemodynamic profile of volatile anesthetics interacts with vasoactive and inotropic therapies commonly employed during cardiac surgery. The net clinical effect therefore reflects not only intrinsic pharmacological properties but also integration within multimodal anesthetic and perfusion strategies [51,75,76]. Understanding these interactions is essential when interpreting clinical studies comparing volatile-based anesthesia with total intravenous techniques and when tailoring anesthetic selection to individual patient physiology. This combination of biological and physiological actions distinguishes volatile anesthetics from purely hypnotic agents and reinforces their potential relevance in contemporary, individualized cardiac anesthetic management.

### 3.4. Clinical Evidence in Cardiac Surgery

#### 3.4.1. Myocardial Protection

Early clinical investigations reported that volatile anesthetic-based regimens were associated with reduced postoperative release of myocardial injury biomarkers, improved early ventricular function and lower requirements for inotropic support. These findings were consistent with experimental evidence of attenuated ischemia–reperfusion injury and supported the hypothesis of anesthetic-induced cardioprotection [10,11,12,13,15,16,18,20,21,22,77,78,79,80,81]. However, subsequent randomized trials and larger cohort studies have yielded variable results, with some confirming reductions in biochemical markers while others demonstrated no significant impact on clinically relevant endpoints such as low cardiac output syndrome, perioperative myocardial infarction or short-term mortality [23,24,25,26,27,28,29,30,31,82]. Differences in cardioplegia strategies, cross-clamp duration, anesthetic dosing and background cardioprotective pharmacotherapy likely contribute to this heterogeneity [83], (Table 2).

#### 3.4.2. Extracardiac Organ Protection

Potential protective effects of volatile anesthetics have also been explored in extracardiac organs exposed to ischemia–reperfusion and inflammatory stress during cardiopulmonary bypass. Several studies suggest attenuation of acute kidney injury incidence or severity, possibly through modulation of renal microcirculatory flow and tubular mitochondrial stress responses [12,15,86]. Neuroprotective effects, reflected by reduced postoperative cognitive dysfunction or delirium, have been reported in cardiac surgical cohorts but remain inconsistently demonstrated across studies [29,84,85]. Pulmonary protective effects of volatile anesthetics have been demonstrated in experimental animal models of acute lung injury and ventilator-induced lung injury, mediated by modulation of inflammatory and endothelial signaling pathways. By contrast, direct clinical evidence demonstrating meaningful respiratory protection in adult cardiac surgical population remains limited [30,87,88]. Finally, attenuation of postoperative hepatic enzyme release has been reported in patients receiving volatile anesthetics during coronary surgery, suggesting possible modulation of hepatic ischemia–reperfusion stress responses, although confirmatory large scale clinical data remains scarce [15].

#### 3.4.3. Volatile Anesthesia Versus Total Intravenous Anesthesia

Comparative studies between volatile anesthetic-based anesthesia and total intravenous anesthesia have been central to clinical debate. While some randomized trials and meta-analyses report modest benefits in myocardial protection and early postoperative recovery with volatile agents [10,11,13,77], others fail to confirm superiority over modern intravenous regimens, particularly in centers employing optimized cardioplegia and perioperative bundles [31,81,82]. Contemporary evidence therefore suggests that any protective advantage of volatile anesthetics is context-dependent, influenced by procedural complexity, patient vulnerability and integration within broader perioperative management strategies. Collectively, clinical data indicate biological plausibility and potential benefit but do not yet support universal superiority of volatile anesthesia in cardiac surgery [30]. These observations highlight the need for refined patient stratification and standardized anesthetic protocols in future trials.

### 3.5. Volatile Anesthetics and Cardiopulmonary Bypass

Cardiopulmonary bypass represents a unique biological and pharmacological environment in which circulating blood is exposed to artificial surfaces, non-physiological flow patterns and controlled hypothermia, collectively triggering systemic inflammatory and coagulation disturbances [5,89]. Within this setting, volatile anesthetics interact not only with myocardial and vascular tissues but also with blood elements and endothelial interfaces, potentially modulating the global host response to extracorporeal circulation. Experimental and clinical studies suggest that volatile anesthetics attenuate complement activation, cytokine release and leukocyte–endothelial adhesion during cardiopulmonary bypass [5]. These effects may contribute to preservation of microcirculatory perfusion and reduction of capillary leak, thereby influencing postoperative pulmonary and renal function [13]. Volatile agents have also been shown to modulate platelet activation and fibrinolytic balance, with potential implications for perioperative bleeding and transfusion requirements, although available evidence remains inconsistent and influenced by confounding surgical variables [59,60,90]. Technical aspects of volatile anesthetic delivery during cardiopulmonary bypass have evolved substantially. Modern vaporizer integration into extracorporeal circuits allows stable anesthetic concentration and controlled uptake, minimizing historical concerns regarding circuit adsorption and unpredictable pharmacokinetics. Nevertheless, interindividual variability in anesthetic uptake during hypothermia and altered pulmonary circulation continues to affect target concentration achievement, underscoring the importance of vigilant monitoring [91,92,93,94,95,96,97]. The interaction between volatile anesthetics and cardiopulmonary bypass therefore extends beyond myocardial protection, encompassing systemic inflammatory modulation, coagulation dynamics and practical considerations of drug delivery. From a practical standpoint, implementation of volatile anesthesia in cardiac surgery requires continuity of anesthetic delivery across all operative phases. During pre-and post-bypass periods, end-tidal anesthetic concentration monitoring reflects alveolar uptake and guides titration of hypnotic depth. During CPB, volatile agents are administered through dedicated vaporizers integrated into the extracorporeal circuit and real-time monitoring of agent concentration in the membrane oxygenator exhaust gas verifies effective anesthetic transfer. Maintenance of stable anesthetic exposure throughout ischemic and reperfusion phases is essential both for adequate hypnosis and for the consistency of potential cytoprotective effects [93,94,95,96,97].

Contemporary practice patterns further illustrate the pragmatic dimension of anesthetic selection in cardiac surgery. A recent survey conducted among members of the Society of Cardiovascular Anesthesiologists in the United States reported that isoflurane was the most frequently selected primary volatile agent across pre-bypass (57%), bypass (62%) and post-bypass (50%) phases. Sevoflurane represented the second-most commonly selected agent in all operative periods. Ease of use was identified as the most frequently cited reason for administering isoflurane and sevoflurane. These findings underscore that, beyond mechanistic considerations, familiarity, workflow integration and technical simplicity remain influential determinants of anesthetic choice in routine clinical practice [98].

### 3.6. Safety, Practical Considerations and Sustainability

Beyond mechanistic and clinical efficacy, the integration of volatile anesthetics into contemporary cardiac anesthesia requires the consideration of safety, feasibility and environmental sustainability (Table 3). From a patient safety perspective, volatile anesthetics have a well-established pharmacological profile, with predictable elimination and limited risk of drug accumulation, particularly relevant in prolonged cardiac procedures. Occupational exposure risk for operating room personnel has markedly decreased with modern scavenging systems, although continuous vigilance remains necessary, especially during cardiopulmonary bypass circuit vaporized integration [91]. Practical implementation also entails logistical and economical considerations. Delivery of volatile anesthetics during cardiopulmonary bypass requires dedicated vaporizers, real-time monitoring of volatile agent concentration in the membrane oxygenator exhaust gas to verify anesthetic transfer and staff familiarity with extracorporeal circuit integration [94,99]. These factors may influence institutional preference for total intravenous anesthesia in certain fast-track or resource-limited settings, despite potential biological advantages of volatile agents. In parallel, environmental sustainability has emerged as a relevant dimension of anesthetic choice. Volatile anesthetics contribute to generate greenhouse gas emissions, with desflurane exhibiting a particularly high global warming potential [100,101,102,103,104,105].

In this context, regulatory policies are beginning to directly shape anesthetic practice: from 1 January 2026, the use of desflurane as inhalational anesthetic is prohibited in the European Union, except in the case of documented medical necessity, owing to its exceptionally high global warming potential, exceeding 2500 times that of CO_2_ [106]. Among currently available volatile agents, sevoflurane exhibits the lowest global warming potential, further supporting its preferential role within environmentally sustainable anesthetic strategies [107].

It should be acknowledged, however, that the climate impact of volatile anesthetics remains an area of scientific debate. Some authors have argued that direct comparisons between volatile anesthetic emissions and carbon dioxide equivalents may oversimplify atmospheric modeling assumptions and overestimate their effective contribution to global warming. In particular, it has been suggested that when contextualized within the broader spectrum of anthropogenic greenhouse gas emissions, the relative climate impact of anesthetic gas emissions is quantitatively small. These considerations highlight the importance of rigorous atmospheric science interpretation while maintaining transparency regarding environmental metrics used in healthcare sustainability policies [108,109]. Growing awareness of healthcare-associated environmental impact has prompted calls for the judicious use of inhalational agents, optimization of fresh gas flows and consideration of agent selection within broader institutional sustainability policies. Balancing potential-centered benefits against environmental responsibility represents an increasingly important aspect of contemporary perioperative decision making.

## 4. Discussion

**Figure 1:**
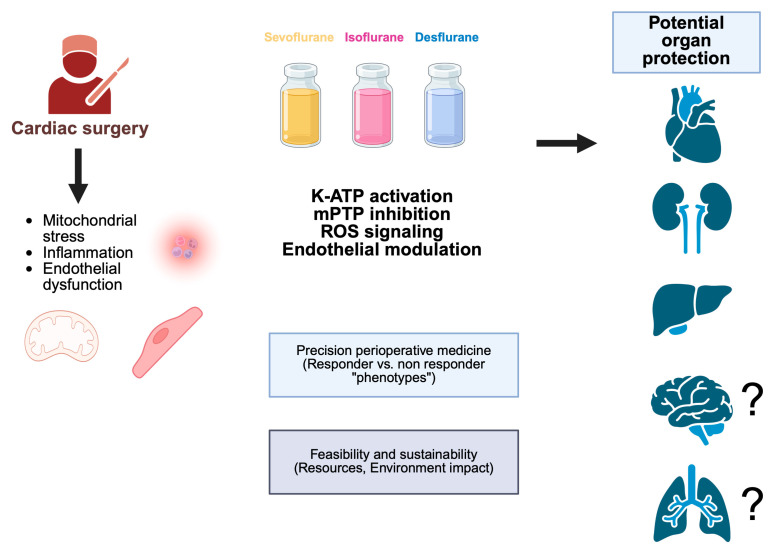
Integrated mechanistic and clinical framework of volatile anesthetic use in cardiac surgery. K-ATP: mitochondrial ATP-sensitive potassium channel; mPTP: mitochondrial permeability transition pore; ROS: reactive oxygen species. Created in BioRender. Pirri, C. (2026) https://BioRender.com/y2s294o.

The recognition that anesthetic management may influence perioperative organ vulnerability has progressively reshaped the traditional view of anesthesia as a purely supportive component of cardiac surgery. Volatile anesthetics occupy a distinctive position within this evolving paradigm, supported by the extensive experimental evidence demonstrating activation of the mitochondrial and endothelial protective pathways that mirror endogenous ischemic preconditioning [47,50,51], (Figure 1). These mechanistic foundations provide a coherent biological rationale for anticipating clinically meaningful attenuation of ischemia–reperfusion injury and systemic inflammatory responses during cardiopulmonary bypass [34,35,45]. Nonetheless, translation of these molecular effects into consistent improvements in patient-centered outcomes has remained challenging. Clinical investigations have yielded heterogeneous findings, reflecting differences in surgical complexity, cardioplegia techniques, anesthetic dosing strategies and baseline patient risk. Moreover, contemporary cardiac surgical practice increasingly incorporates optimized myocardial protection, controlled reperfusion protocols and multimodal anti-inflammatory measures, potentially limiting the incremental benefit attributable to anesthetic choice alone [30,31,110]. In this context, the absence of universal superiority of volatile-based anesthesia should not be interpreted as lack of biological efficacy, but rather as evidence of complex interactions among multiple perioperative protective interventions. The emerging framework of precision perioperative medicine offers an opportunity to reconcile these observations. Interindividual variability in mitochondrial resilience, inflammatory responsiveness, endothelial integrity and genetic background likely determines susceptibility to ischemia–reperfusion injury and responsiveness to anesthetic-induced cytoprotection [49,56]. Identification of responder phenotypes through molecular profiling or functional biomarkers may enable targeted application of volatile anesthetic strategies to patients most likely to benefit, while avoiding unnecessary exposure in low-yield scenarios. Such an approach aligns anesthetic selection with broader trends toward individualized risk stratification and tailored perioperative care. At the same time, anesthetic decision-making must be integrated with practical and societal considerations, including feasibility of volatile delivery during cardiopulmonary bypass, institutional resources and growing environmental sustainability imperatives. Practical considerations include the availability of integrated vaporizers within extracorporeal circuits, institutional familiarity with volatile delivery during cardiopulmonary bypass, real-time monitoring of anesthetic concentrations at the oxygenator exhaust and coordinated training of anesthesia and perfusion teams. Resource allocation, workflow organization and the need to ensure uninterrupted anesthetic exposure across systemic and reperfusion phases may therefore influence the feasibility and consistency of volatile-based strategies. From a societal perspective, increasing attention to healthcare-associated carbon footprint, regulatory constraints and institutional sustainability policies may progressively shape anesthetic selection. In high-resource environments, decision-making increasingly reflects not only patient-centered outcomes but also environmental stewardship and cost-awareness. The integration of these dimensions underscores the complexity of contemporary anesthetic planning in cardiac surgery and reinforces the need for balanced, context-sensitive strategies.

These factors increasingly influence anesthesia practice in high-resource surgical settings and underscore the need to balance potential patient-level benefit with responsible healthcare stewardship [98]. Recent international guidelines in cardiac anesthesia and perioperative organ protection currently refrain from issuing definitive recommendations regarding anesthetic choice for cardioprotection, reflecting persistent uncertainty in the field [111].

Taken together, current evidence supports a biologically plausible and potentially clinically relevant role for volatile anesthetics in cardiac surgery, while underscoring the need for refined patient stratification, standardized protocols and integration with multimodal perioperative protective strategies [56,112,113,114].

## 5. Conclusions

Volatile anesthetics exert multifaceted biological and physiological effects that extend beyond hypnosis, encompassing mitochondrial cytoprotection, modulation of inflammatory and endothelial responses and distinct hemodynamic and metabolic profiles. These properties provide a compelling mechanistic basis for their proposed role in attenuating ischemia–reperfusion injury and perioperative organ dysfunction in cardiac surgery. Nevertheless, clinical evidence remains heterogeneous and universal superiority over contemporary intravenous anesthetic strategies has not been conclusively demonstrated. Within the evolving landscape of precision perioperative medicine, volatile anesthetics may ultimately find their greatest value as targeted component of individualized organ protection strategies rather than as uniform default agents. Balancing mechanistic potential, clinical efficacy, feasibility and environmental sustainability will be essential to define their rational integration into modern cardiac anesthetic practice.

## 6. Future Directions

Future research should prioritize improved characterization of patient-specific biological factors that may influence responsiveness to volatile anesthetic-mediated cytoprotection. Integration of emerging genomic, proteomic and metabolomic approaches with perioperative outcome data could, in the future, support development of predictive frameworks for anesthetic responder phenotypes; however, current costs, technical complexity and limited clinical validation restrict these tools primarily to research settings and trial enrichment. Large, pragmatic, randomized trials employing standardized cardioplegia protocols, controlled anesthetic dosing and contemporary perioperative care bundles remain necessary to clarify the magnitude of clinical benefit in defined patient subgroups. Further investigation into optimal timing, duration and concentration of volatile anesthetic exposure during ischemia and reperfusion is warranted, alongside evaluation of potential interactions with pharmacological cardioprotective agents and mechanical perfusion strategies. Finally, future anesthetic research should incorporate environmental impact assessments and cost-effectiveness analyses to guide sustainable and equitable implementation of perioperative care.

## Figures and Tables

**Table 1:** Cellular and molecular pathways involved in volatile anesthetic-induced cytoprotection.

Pathway	Target	Effect of Volatile Anesthetics
Mitochondrial K-ATP [47,51]	Mitochondrial membrane	Stabilization, ↓ Ca overload
mPTP opening [35]	Mitochondrial pore	Delayed opening
ROS signaling [53]	Cytosolic kinases	Trigger of preconditioning
Endothelial NO [58]	Microcirculation	↓ leukocyte adhesion
Glycocalyx [62]	Endothelium	↓ shedding

K-ATP: mitochondrial ATP-sensitive potassium channel; mPTP: mitochondrial permeability transition pore; ROS: reactive oxygen species; NO: nitric oxide; Down arrow: decrease.

**Table 2:** Clinical evidence on volatile anesthetic-based strategies and perioperative outcomes in cardiac surgery.

Reference	Study Design/Population/Sample Size	Anesthetic Comparison	Primary Endpoints	Main Reported Findings	Authors’ Interpretation (Endpoint-Specific)
Li and Yuan, 2015 [10]	Meta-analysis; cardiac surgery N = 1646 (15 trials)	Sevoflurane vs. Propofol	Myocardial injury biomarkers (cTnI)	Sevoflurane associated with lower postoperative cTnI levels and lower incidence of late adverse cardiac events	Favors Sevoflurane for biomarker attenuation
Malagon et al., 2005 [12]	Randomized pediatric cardiac surgery study (congenital heart defect) N = 90	Sevoflurane vs. alternative anesthetics (propofol, midazolam)	Cardiac troponin T release	Equivalent myocardial protection across anesthetic regimens. cTnT was elevated in all 3 groups throughout the study period	Neutral
Julier et al., 2003 [13]	Double blinded, placebo-controlled, RCT; CABG N = 72	Sevoflurane preconditioning vs. placebo (oxygen–air mixture)	Myocardial and renal dysfunction biomarkers (brain natriuretic peptide, cystatin C)	Significant reduction in biochemical markers of myocardial and renal injury	Favors Sevoflurane on organ injury biomarkers
Lorsomradae et al., 2006 [15]	Prospective, double-blind RCT; CABG N = 320	Sevoflurane vs. Propofol	Hepatic and renal injury biomarkers (SGOT, SGPT, LDH, creatinine)	Lower postoperative hepatic injury markers with sevoflurane; no difference in creatinine	Favors Sevoflurane on hepatic biomarkers; neutral on renal function
Bignami et al., 2009 [16]	Multicenter observational comparative study; CABG N = 34,310	Volatile vs. non-volatile anesthesia	Risk-adjusted mortality	Volatile anesthetics associated with lower risk-adjusted mortality	Observational signal favoring volatiles
Dharmalingam et al., 2021 [17]	Prospective RCT; CABG with CPB N = 18	Sevoflurane vs. Isoflurane	Oxidative stress markers and nitric oxide levels; myocardial injury biomarkers (CK-MB)	Sevoflurane group showed reduced oxidative stress parameters and improved nitric oxide modulation compared to isoflurane; lower postoperative CK-MB levels observed	Favors Sevoflurane on biochemical markers of oxidative stress and myocardial injury; mechanistic support without hard clinical outcome differences.
El Dib et al., 2017 [18]	Systematic review; CABG on/off pump N = 6105 (58 trials)	Inhalational vs. intravenous anesthesia	Mortality (180–365 days) and inotropic/vasoconstrictor support	Sevoflurane associated with reduced intermediate-term mortality and inotropic and vasoconstrictor requirement	Favors Sevoflurane for selected outcomes (lower 180–365 days mortality, lower inotropic and vasoconstrictor support)
Zhang et al., 2023 [20]	Meta-analysis; off-pump CABG N = 703 (14 RCTs)	Volatile anesthesia vs. Propofol	Myocardial injury biomarkers (cTnI, cTnT) and major adverse events	Reduced Troponin release with Sevoflurane; no difference in secondary outcomes (postoperative mechanical ventilation time, length of ICU-stay and mortality)	Favors volatiles on biomarkers; neutral on hard outcomes
Landoni et al., 2013 [21]	Bayesian network meta-analysis; cardiac surgery N = 38 RCTs	Volatile anesthetics vs. TIVA	Mortality	Volatile anesthetics suggested to reduce mortality; authors call for confirmation in large RCTs	Hypothesis-generating signal favoring volatiles
Barelli et al., 2025 [22]	Randomized pediatric clinical trial N = 66	Sevoflurane vs. TIVA	Troponin I and renal function (urine output and serum urea levels)	No difference in troponin release; possible renal functional benefit	Neutral on myocardial biomarker; exploratory renal signal
Soro et al., 2012 [23]	Double-blind randomized trial; CABG N = 75	Sevoflurane vs. Propofol (intra and postoperative sedation)	Myocardial injury biomarkers and hemodynamic outcomes	No significant intergroup differences in biomarker release or perioperative events	Neutral on biomarkers and clinical endpoints
Varsha et al., 2024 [24]	Prospective randomized blinded study; CABG with CPB N = 72	Sevoflurane vs. Propofol TIVA	Postoperative delirium and cognitive dysfunction	Lower incidence of delirium and postoperative cognitive dysfunction in TIVA group	Favors TIVA for neurocognitive endpoints
Garg et al., 2025 [25]	Prospective RCT; on pump CABG N = 50	Volatile anesthesia vs. Propofol-based TIVA	Inflammatory response (IL-6) and early cognitive recovery	No difference in IL-6 response; faster emergence and improved early cognitive scores with TIVA	Neutral on inflammation; favors TIVA for early neurocognitive recovery
Ren et al., 2019 [26]	Systematic review and meta-analysis; valve surgery N = 962 (13 RCTs)	Inhalational vs. intravenous anesthesia	Survival and major complications	No superiority of inhalational anesthesia; evidence judged insufficient for definitive conclusions	Neutral with limited certainty
Deng et al., 2024 [28]	Multicenter RCT, adult cardiac surgery N = 3123	Volatile anesthesia vs. Propofol-based TIVA	Major postoperative complications and 30 days mortality	No difference in overall clinical effectiveness between anesthetic strategies	Neutral on composite clinical outcomes
Jiang et al., 2023 [29]	Randomized controlled trial; valve surgery and/or CABG N = 684	Volatile anesthesia vs. Propofol-based TIVA	Postoperative delirium	No significant difference in delirium incidence or secondary outcomes	Neutral on neurocognitive outcomes
MYRIAD Trial, 2019 [31]	Multicenter RCT; elective CABG N = 5400	Volatile anesthesia vs. Propofol-based TIVA	One-year all- cause mortality	No significant difference in one-year mortality between groups	Neutral on hard clinical outcomes
Jiao et al., 2019 [82]	Meta-analysis and trial sequential analysis; CABG N = 14,387 (89 RCTs)	Volatile anesthesia vs. TIVA	Operative mortality and safety outcomes	No reduction in mortality or major safety endpoints; cumulative evidence inconclusive	Neutral and statistically inconclusive
Schoen et al., 2011 [84]	Prospective double-blind RCT; on pump CABG N = 128	Sevoflurane-based anesthesia vs. propofol-based TIVA	Postoperative cognitive function	Patients in the sevoflurane group has better performance on multiple cognitive stress compared with propofol; no differences in organ dysfunction or general clinical outcomes	Favors Sevoflurane for short-term postoperative cognitive function; neutral on broader clinical outcomes
Han et al., 2024 [85]	Systematic review and meta-analysis; adult cardiac surgery; N = 10 RCTs	Volatile anesthesia vs. propofol-based TIVA	Postoperative cognitive function	No significant difference in postoperative cognitive function	Neutral

CABG: coronary artery bypass grafting; CPB: cardiopulmonary bypass; TIVA: total intravenous anesthesia; RCT: randomized controlled trial; cTnI: cardiac troponin I; cTnT: cardiac troponin T; CK-MB: creatine kinase-myocardial band; IL-6: interleukin 6; SGOT (AST): serum glutamic oxaloacetic transaminase; SGPT (ALT): serum glutamic pyruvic transaminase; LDH: lactate dehydrogenase; ICU: intensive care unit.

**Table 3:** Environmental impact of commonly used volatile anesthetics.

Agent	GWP100 (CO_2_ Equivalents)	Atmospheric Lifetime	Regulatory Status (Illustrative Policy Example)
Desflurane	~2540	~14 years	Routine clinical use restricted from 2026 (EU) *
Isoflurane	~510	~3 years	No current restriction
Sevoflurane	~130	~1 year	No current restriction

GWP100: global warming potential over a 100-year time horizon, expressed relative to CO_2_; CO_2_: carbon dioxide; EU: European Union. * Except for documented medical necessity (EU-F Gas Regulation 2024).

## Data Availability

No new data were created or analyzed in this study. Data sharing is not applicable to this article.

## Abbreviations

The following abbreviations are used in this manuscript:

CABG	Coronary artery bypass grafting
CPB	Cardiopulmonary bypass
TIVA	Total intravenous anesthesia
K-ATP	Mitochondrial ATP-sensitive potassium channel
